# A review of the impact on the ecosystem after ionizing irradiation wildlife population

**DOI:** 10.1080/09553002.2020.1793021

**Published:** 2020-07-24

**Authors:** Georgetta Cannon, Juliann G. Kiang

**Affiliations:** a Radiation Combined Injury Program, Scientific Research Department, Armed Forces Radiobiology Research Institute, Uniformed Services University of the Health Sciences, Bethesda, MD, USA; b Department of Pharmacology and Molecular Therapeutics, Uniformed Services University of the Health Sciences, Bethesda, MD, USA; c Department of Medicine, Uniformed Services University of the Health Sciences, Bethesda, MD, USA

**Keywords:** Radiation, ecosystem, wildlife, population, DNA damage, apoptosis

## Abstract

**Purpose:**

On 26 April 1986, reactor 4 at the Chernobyl power plant underwent a catastrophic failure leading to core explosions and open-air fires. On 11 March 2011, a combination of earthquake and tsunami led to a similar disaster at the Fukushima Daiichi power plant. In both cases, radioactive isotopes were released and contaminated the air, soil and water in a substantial area around the power plants. Humans were evacuated from the immediate regions but the wildlife stayed and continued to be affected by the ongoing high radiation exposure initially and later decayed amounts of fallout dusts with time. In this review, we will examine the significant effects of the increased radiation on vegetation, insects, fish, birds and mammals.

**Conclusions:**

The initial intense radiation in these areas has gradually begun to decrease but still remains high. Adaptation to radiation is evident and the ecosystems have dynamically changed from the periods immediately after the accidents to the present day. Understanding the molecular mechanisms that allow the adaptation and recovery of wildlife to chronic radiation challenges would aid in future attempts at ecosystem remediation in the wake of such incidents.

## Introduction

Twenty-one years later after the Chernobyl power plant explosion, various isotopes of plutonium, strontium-90, americium-241, and cesium-137 were still detected at high levels causing adverse biological effects across the nearby areas (Voitsekhovych et al. 2007). Wildlife continued to be exposed to substantial radiation doses after humans were evacuated from these areas. The half-life of cesium-137 is approximately 30 years and it decays by *β* emission to a metastable isomer of barium-137. The half-life of barium-137 isomer is 2 minutes. Subsequently, the metastable isomer emits *γ* radiation and becomes ground state barium (Baum et al. 2002). Food or water contaminated with cesium-137 that are ingested lead to internal *β* and *γ* radiation doses in addition to external radiation doses. The half-life of cesium-134 is about 2 years. Cesium-134 emits *β* particles. The half-life of strontium-90 is approximately 29 years. Strontium-90 emits pure *β* radiation. Most of the plutonium isotopes emit *α* particles, which are ionizing and harmful, but have a short penetration distance. The half-life of plutonium-241 is approximately 14 years. It emits *β* radiation to become americium-241. The half-life of americium-241 is 432 years, and it emits *α* particles to become neptunium-237, with a by-product of *γ* emissions (Baum et al. 2002). This is the composition of radiation released and retained in the soil, water and air across the Chernobyl landscape. In addition to external radiation exposure, ingestion of contaminated food and water by wildlife occurred from the beginning of the disaster and continues to the present.

In contrast to the Chernobyl disaster, for Fukushima, a relatively smaller area of land and water was contaminated with cesium-134 and cesium-137 with radioactive noble gases released to air. The cesium isotopes are expected to be responsible for 98% of the next 30-year-cumulative radiation dose in the surrounding area of Fukushima (Imanaka et al. 2015).

Many scientific studies were begun at once after the Chernobyl disaster (Copplestone et al. 2008; UNSCEAR 2011) and mathematical models used for predicting environmental impacts based on the data were adopted to predict how the Fukushima disaster would affect the ecosystem (Aliyu et al. 2015; Bréchignac et al. 2016). Unfortunately, studies on the impact of chronic radiation to the physiology of the remaining organisms in the environment are limited. Changes in the food chain are surely inevitable, but relatively few studies have been focused on the ecosystem as a whole exposed to chronic medium to low levels of radiation. Many laboratory experimental animal models, such as mice, rats, dogs, pigs, and nonhuman primates, indicate that ionizing radiation is a poly-traumatic hit resulting in multi-organ injury to individuals (Kiang and Olabisi 2019). Presumably ionizing radiation produces similar results in wild animals and plants, and over time to the ecological network as a whole particularly when the hits keep on coming for an extended time.

In this brief review, we summarize observations with insects, plants, birds, fish, and mammals chronically exposed to radiation released in contaminated soil, water and air. Observations of adaptation to radiation is included. Molecular mechanisms underlying responses of the wildlife to radiation as well as identification of biomarkers for evaluating changes in the ecosystem will be discussed and recommended.

### Effects of radiation on insects

In 2009, around 23 years after the disaster, the insect abundance within the Chernobyl zone during 2006–2008 versus insects from other forested locations around Chernobyl, Ukraine and Belarus were investigated (Moller and Mousseau 2009). The study observed 298 bumble-bees with 72.6% of them being *Bombus terrestris*. The abundance of bumble-bees decreased in the radiation-contaminated areas in a radiation dose-dependent fashion. The reductions also depended on environmental elements such as year, temperature, wind and habitat where bumble-bees were collected. Three hundred eighty-nine butterflies with 36.6% of them being *Aporis Crataegi* were collected. As with the bumble-bees, the butterfly population was significantly reduced in the radiation-contaminated area depending on the severity of radiation contamination. The time of the day, wind and type of habitat introduced variance into the population reductions. Three hundred and five grasshoppers were collected around Chernobyl, Ukraine and Belarus. The grasshopper population was significantly more diminished if radiation levels were higher. Dragonflies were investigated as well. One hundred and five dragonflies were collected. The population significantly declined based on the severity of radiation doses. The results were varied and correlated with time of day, date, habitat, temperature, and cloud cover. Finally, seven hundred seventy-five spider webs were observed. The spider population appeared to decrease depending on radiation severity, year, temperature and wind. At this time, most radiation around Chernobyl was in the topmost soil, where most insects lived (Shestopalov 1996). For example, butterfly eggs, larvae and pupae developed in the radiation-contaminated soil layer. Pollinator abundance was reduced, thereby, affecting plant fecundity and seed set. This impacted the insect habitat (Proctor et al. 1996). Spiders were one of the key players in predation (Wise 1993) and the attenuation of their abundance led to increases in abundance of other insects (Snyder and Wise 2001). Shortly after the Chernobyl disaster, studies performed in the 30Km impact zone have demonstrated that 30-Gy doses did not directly affect adult animals in the soil and litter, but impacted their eggs and juveniles. After two to two and half years, the significant differences between insect populations in the contaminated areas and uncontaminated areas were not found any more (Krivolutzkii and Pokarzhevskii 1992). This may be because insects from uncontaminated areas migrated into the contaminated areas, leading to the recovery of the soil animal population (Krivolutzkii and Pokarzhevskii 1992). These results were different from the 2009 results which show the effect of long-term chronic radiation (Moller and Mousseau 2009).

Comparing the observations among bumble-bees, butterflies, grasshopper, dragonflies and spider webs, Table 1 indicates the relationship between insect abundance and environmental elements. All five species of insects are sensitive to radioactive contamination. All except spiders (as indicated by web production) were also sensitive to other changes in their habitats. The habitat temperature varied along with cloud cover, wind, time of day, date, and year. Most interestingly, the population of grasshoppers was affected by cloud cover and date but not wind and year, whereas populations of bumble-bees and spider webs were influenced by all these factors. The results indicate a holistic approach is necessary to understand environmental impacts. It should be noted that different sites which had the same radiation levels might have differing habitat (i.e. ground cover), soil composition, or water level/access. Since the collections took place over 3 years, temperature, cloud cover, wind, time of day, actual date and year were also considered and tested as potential confounding variables.

In general, adult insects are believed to be resistant to radiation. These insects were significantly affected by radiation possibly because of the ingestion of a combination of plutonium, strontium-90, americium-241, and/or cesium-137 leading to internal contamination with *α*, *β* and *γ* combined radiation in these areas (Voitsekhovych et al. 2007). The eggs and larva forms are also more sensitive to radiation in general and some grow in close contact with the contaminated ground (Bonisoli-Alquati et al. 2018).

Besides the impact of environmental elements on the insect population, persistent genetic damage was detected. Hancock and colleagues (Hancock et al. 2019) investigated the relationship between historic radiation doses and the genetic damage found in populations of Drosophila (*Drosophila melanogaster*) around the Chernobyl Nuclear power Plant. For earlier generations of Drosophila living in the radioactive contaminated locations, the sex-linked recessive lethal (SLRL) rates appeared to correlate with the dose in a linear, non-threshold relationship. The later descendent generations exhibited a radio-adaptive-like response with a plateau in SLRL frequencies. This adaptation is thought to be important for future Drosophila survival through competition and selection, or evolution.

Shortly after the Fukushima disaster, pale grass blue butterflies (*Pseudozizeeria maha*), whose populations were over-wintering as larvae at time of the accident were examined. This species lives concurrently with humans as well as other wildlife and is multivoltine, taking about one month per generation. The first-voltine adults, the F_1_ generation, and the F_2_ generation were collected to study their wing size, half-closing time, and morphological malformations including wings, abdomen, legs, eyes, palpi, and antennae (Hiyama et al. 2012). Some of first-voltine adults in the Fukushima area, collected in May 2011, manifested relatively mild abnormalities. However, the F_1_ offspring from the first-voltine females exhibited more severe abnormalities, which continued to be seen in the F_2_ offspring. Adult butterflies collected in September 2011 showed more severe abnormalities than those collected in May 2011. Most importantly, similar abnormalities were experimentally confirmed when butterflies from noncontaminated areas were exposed to artificial external and internal radiation at a low dose. The authors concluded that the physiological and genetic damage observed in the butterflies resulted from exposure to radionuclides from the Fukushima Nuclear Power Plant. This species was also monitored from 2011–2013 (Taira et al. 2015). The distribution of abnormality and mortality rates continued to be radiation dose-dependent. Abnormality and mortality rates reached a peak level primarily in the fall of 2011 and returned to normal levels afterwards. Early radiation exposure had a higher impact than later radiation exposure. Radiation effects were accumulated trans-generationally over a specific period. The population regained normality relatively quickly after about 15 generations within 3 years. They concluded that even low doses induced morphological abnormalities and death for some butterflies of this species, whereas other individuals were not affected, at least morphologically. This variable sensitivity implied the possibility of radiation resistance or adaptive evolution. However, the data observed from 298 haploid genomes of *Drosophila melanogaster* collected in 2012 showed no chromosomal inversions, and 2006 genomes collected in 2013 contained only 2 chromosomal inversions. The authors claimed no convincing data to suggest that the chronic radiation released in the Fukushima disaster caused genetic damage (Itoh et al. 2018). This result in Fukushima was different from that found in Chernobyl where chronic radiation increased the SLRL rate in Drosophila (Hancock et al. 2019). The chronic dose rates in Fukushima were much lower than the ones in Chernobyl which may explain why no genetic damage was found in this case. However, molecular level changes in Chernobyl’s and Fukushima’s wildlife are not yet fully understood. Further studies should be explored.

### Effects on plant life

Seed set, seeding consumption, seed dispersal and maintenance of plant communities in the ecosystem are impacted by co-living animals. Moller et al. (Moller et al. 2012) studied fruit trees such as rowan (*Sorbus aucuparia*), pear (*Pyrus communis*), and apple (*Malus domestica*), and bushes such as European cranberry bush (*Viburnum opulus*), twisting-wood (*Viburnum lantana*), and wild rose (*Rosa rugose*) along with butterflies and bumble-bees in the area of Chernobyl. In the areas with high radiation contamination, they found insect-pollinated fruit plants produced fewer fruits and this correlated with the local reduction in pollinating insects and with the generally smaller size of fruit trees. Lower fruit abundance led to lower levels of fruit-eating birds thus further limiting seed dispersal. These observations suggest that the abundance of frugivores, pollinating insect populations, and fruit production were directly associated with radiation levels. In other words, the number of fruit-bearing trees was low when the level of radiation was high, while the fruit sets were low when pollinating insect populations was low due to high radiation levels. The results taken together suggest that the close interplay among many species and between animals and plants is strongly affected by chronic radiation. Direct killing of the fruit trees by high radiation resulting in the overgrowth of other plants years later should be also be considered.

During the period of 1986–1991, after the Chernobyl disaster, pine trees (*Pinus silvestris*) within a 10-km radius zone from the Chernobyl Power Plant were significantly hurt (Arkhipov et al. 1994). Radiation doses at 10–60 Gy, 1–10 Gy and 0.1–1 Gy resulted in high, medium and low damage to pine trees, respectively. Radiation doses more than 60 Gy led to a massive fatality without recovery or regeneration of pine trees since 1987. The radiation-exposed pine trees dried-up and/or burned down. Those dying trees were infested by pathogenic insects. Pine trees exposed to radiation doses less than 0.1 Gy did not display any visible injury. Pine trees appeared to be capable of repairing the medium and low degrees of injury (Arkhipov et al. 1994), suggesting their regeneration ability remains.

It was thought that radiation would cause more intense stress to plant growth at certain stages. Boratynaki and colleagues (Boratyński et al. 2016) reported that they used 660 seeds collected from 33 wild carrots (*Daucus carota*) from various regions near Chernobyl (that significantly varied in radioactive contamination) to conduct a common garden experiment in an uncontaminated greenhouse. They found that the higher the level of radiation received by the maternal plants was, the longer the timing and the slower the rates of seed germination were. Radiation exposure also prolonged leaf production. Again, the higher the radiation dose was, the longer leaf production took. The results suggest that radiation slows down the cell proliferation capacity and that is consistent with what we have observed in recovery of mouse skin wound after irradiation (Kiang et al. 2010).

### Effects on birds

When the lens of the eye becomes opaque, the eye’s ability to see is reduced. The lens opacity is known as a cataract and can occur when eyes are exposed to radiation that can be either non-ionizing or ionizing. We would expect cataracts to make free-living animals less fit in a radiation-contaminated environment. Cataracts in free living birds were increased in a dose-dependent manner with the increasing level of background radiation, but cataract development showed no threshold of radiation dose. Birds with opacities in one lens had a greater probability of developing opacities in the lens of the other eye as well, resulting in even poorer vision. The incidence of lens opacities, typical of cataracts, in more than 1100 free-living birds in the Chernobyl region was investigated to elucidate their relation to the background radiation (Moller and Mousseau 2007). The incidence of cataracts was increased when increasing levels of background radiation was detected. The data were analyzed based both on a dichotomous score and on continuous scores of intensities of cataracts. The odds ratio per unit change in the regressor was 0.722 (95% CI 0.648, 0.804). It was lower than odds ratios from investigations of radiation cataracts in humans. The small odds ratio in birds may be driven by increased fatality in birds with cataracts in both eyes, because cataracts impede a bird’s mobility to hunt food, to stay away from predators, to fly safely and even to mate and raise offspring. No increases in incidence of cataracts with increasing age were found, suggesting that yearlings and older birds were similarly affected by radiation-induced cataracts. The authors found a robust inverse correlation between bird abundance and background radiation, suggesting that radiation lowers bird abundance via not only an increase in the probability of cataracts in bird populations, but also via effects on disease susceptibility, food abundance and interactions with other species. As part of the ecosystem, for example, if fruit-eating birds like warblers and thrushes suffered cataracts, which would decrease their ability to find and eat fruits to disperse seeds, there would be soon be fewer fruit-bearing trees available in areas leading to a lower fruit abundance, even after controlling the directly detrimental effects of radiation on fruit trees. This could cause remaining birds to starve to death in an adverse cycle (Moller and Mousseau 2007). Therefore, fruit-eating birds, seed dispersal, fruit trees, fruit abundance, and pathogen invasion are affected not only individually, but also collectively by radiation leading to adverse outcomes that synergize to amplify the detrimental impacts on the ecosystem.

There are no reports of radiation induced cataract occurrence in insects mentioned above or fish discussed below. However, radiation-induced cataracts are not unique to birds. Humans and other mammals are known to develop radiation-induced cataracts (Laskowski et al. 2020). More research in this area with other animals including pigs, cows and nonhuman primates should be considered.

Studies on birds at Fukushima have been more limited but have been shown that both bird species and abundances decrease abruptly since the disaster (Moller et al. 2015), suggesting the low-dose radiation induces a similar outcome as the high-dose radiation does, although the underlying mechanisms for the low-dose radiation and the high-dose radiation may be different. More studies in this regard should be explored.

### Effects on fish

Fish are radiation sensitive. At Chernobyl, fish in the freshwater environment have been highly exposed to radiation since the disaster happened (Lerebours et al. 2018). Little is known about the biological effects of chronic radiation exposure at low level on fish. Lerebours and colleagues collected 142 roach (*Rulilus ruitilus*) and 162 perch (*Perca fluviatilis*) in total from seven lakes in Belarus and Ukraine. Specific activities of americium-241, plutonium-238 — 239 — 240, strontium-90, and cesium-137, index conditions, size and distribution of oocytes, as well as biological and environmental confounding factors in these two fish species were investigated. The results indicated that the general physiological and reproductive health of roach and perch were not affected. Nonetheless, roach appeared to be less effected by radiation than perch. In most radiation-contaminated lakes, numbers of perch exhibited radiation-delayed maturation of gonads and the presence of several undeveloped phenotypes not seen in roach. Perch are a carnivorous fish and as such accumulated more cesium-137 than their prey fish, roach which may account for the differences. The differences may also be due to variations in epigenesis, genetic composition, signal transduction and energy production. The metabolomics approach would probably be able to shed light on why roach are less sensitive to radiation than perch.

The biological effects of the radiological Fukushima disaster on fish are little known, because it is difficult to separate them from the effects of the preceding earthquake followed by tsunami and because most models imply that radioactivity doses seen there are too low to induce biological effects. However, the prohibitions on fishing in the area would have been anticipated to lead to increased populations of target fish, which has not yet happened (Kodama et al. 2018). With all the uncontrolled variables, it is difficult to evaluate how low-dose radiation might chronically impact the ecosystem. With this in mind, studies with low-dose radiation in the laboratory setting should be encouraged.

### Effects on mammals

Pomerantseva and colleagues (Pomerantseva et al. 1997) caught house mice from 1986 to 1994 in radionuclides-polluted regions after the Chernobyl disaster. The dose rates of *γ* radiation on the soil surface ranged from 0.0002 to 2 mGy/h. Using the air-drying method of Evans et al. (1964) for meiotic preparations from mammalian testes, they found reciprocal translocation occurred in these house mice. The frequency of reciprocal translocation in mouse spermatocytes was increased when dose rates were increased. Embryonic lethality was higher in the progeny of male mice caught in 1987 in the most contaminated area compared to data obtained in the less contaminated area. These changes were reduced with time after the disaster. In another study (Shevchenko et al. 1992), among 74 mice caught around the Chernobyl nuclear power station, four mice showed reciprocal translocation heterozygotes but increased embryonic lethality and an increased number of abnormal sperm heads were found. These results suggest that radiation severely harms the reproductive system but that the effects decrease over time perhaps due to the die-off of the most affected families and adaptation of the less affected ones. Similar field studies should be continued to verify whether adaptation appears. The competition and the selection that are fundamental principles of evolution require time. Studies with subjects collected from Chernobyl may now show evidence evolution/adaptation.

Radiation-induced DNA double-strand breaks (DSB) can be detected by measuring phosphorylated H2AX (i.e. *γ*-H2AX) at the DNA break sites, which is a well-established biomarker. *γ*-H2AX is so sensitive to radiation and can be induced even with radiation exposure at 1 mGy (Rothkamm and Lobrich 2003). Cows abandoned at Fukushima were examined for *γ*-H2AX foci in their peripheral circulating lymphocytes. Nakamura and colleagues (Nakamura et al. 2017) collected blood samples from cows (*N* = 70) from every location of the ex-evacuated area in Fukushima and blood samples from cows at radiation-uncontaminated cities (*N* = 8 per region) as a control group. Using immunohistochemistry, they found that the number of *γ*-H2AX foci in peripheral circulating lymphocytes of cows from the area contaminated with radiation was twofold higher compared with the data from the control group. The levels of DNA damage slightly decreased over the 700-day sample collection period. The extent of damage did not appear to be associated with the distance from the accident site, whereas an age-dependent accumulation of DNA damage was apparent. The results suggest that age and time are confounding factors for measuring DNA damage. Notably, such low-dose radiation is sufficient to cause a significantly persistent DNA damage even 700 days later. Whether this persistent DNA damage would result in teratogenic effects should be explored.

When human residents at areas near the Fukushima Daiichi Nuclear Power plant were evacuated, Japanese macaques (*Macaca fuscata*) residing there were also evacuated. Japanese macaques (N = 72) were captured between May 2013 and December 2014 within a 40-kilometer radius of the power plant. The nonexposed (control) group consisted of 23 macaques. They were divided into the immature group (≤1-year old) and the mature group (≥5 years old). The median internal dose-rate was 7.6 *μ*Gy/day (ranging from 1.8 to 219 *μ*Gy/day) and the external dose rate was 13.9 *μ*Gy/day (ranging from 6.7 to 35.1 *μ*Gy/day). White blood cells (WBCs), red blood cells (RBCs), hemoglobin (HGB), hematocrits (HTC) and platelets (PLTs) were measured in their blood samples. Bone marrow samples were used for histopathological analysis. The white blood cell and platelet counts appeared to be low when the internal dose rate in mature macaques was high. Likewise, the myeloid cell, megakaryocyte, and hematopoietic cell counts in the bone marrow samples were low, but adipocyte counts in the bone marrow were high when internal dose-rate in femoral bone marrow of mature macaques was high (Urushihara et al. 2018). The observations are in agreement with the data obtained in mice (Kiang et al. 2010; Kiang and Olabisi 2019), minipigs (Kiang and Smith, unpublished data) and nonhuman primates (Wong et al. 2020). These results suggest that both acute radiation exposure and chronic radiation exposure lead to hematopoietic depletion, which is a typical radiation syndrome and fatal if no proper care was provided. Acute radiation syndrome (ARS) is well characterized (Kiang and Olabisi 2019), but delayed acute radiation effect (DEARE) and long-term radiation impact are not well understood in wildlife. More future effort should be focused on the latter two areas.

### Adaptation to radiation

Radiation intensity in both Chernobyl and Fukushima has decreased with time. Under these conditions, organisms may evolve ways of surviving the radiation stress, also called adapting. Bonisoli-Alquati and colleagues (Bonisoli-Alquati et al. 2018) captured grasshoppers (*Chorthippus albomarginatus*) in the Chernobyl exclusion zone and raised their offspring in a common garden. They found that offspring that matured quicker than average grasshoppers contained more DNA damage. Faster growth rates cause elevated oxidative stress and subsequent injury, but the grasshoppers may gain time to reach adulthood and to reproduce before the radiation released from the environment kills them. The result suggests the presence of an adaption by maintaining the population at the expense of more DNA damage to the individual.

Similar results may be found in other species. For example, pale grass blue butterflies (*Zizeeria maha*) that survived in Fukushima at the low radiation dose may be examples of adaptation (Taira et al. 2015). Also, the frequency of sex-linked recessive lethal genes in *Drosophila melanogaster* was mitigated in later descent generations compared to the data observed with earlier generations (Hancock et al. 2019). These examples reinforce the idea that radiation adaptation can occur. Table 2 lists more studies describing data from insects, plants and mammals in support of adaptation.

It is possible that resistance to acute radiation and to chronic radiation may involve different underlying mechanisms. Studying the chronic radio-resistance phenomenon is complex in the field where environmental elements such as wind, cloud cover, temperature, seasonal changes, interspecies competition, and habitat (Table 1) are not controllable. On the other hand, it is almost impossible to design experiments with consideration of all parameters mentioned above under control in the laboratory. Both all encompassing (but messy) field studies and controlled (but limited) laboratory studies are necessary. A system biology approach is needed (Shuryak 2020).

## Molecular mechanisms

Almost no underlying molecular mechanisms for radiation-induced biological changes are defined in wildlife. Offspring of grasshoppers (*Chorthippus albomarginatus*) captured in the Chernobyl exclusion zone and raised in a common garden manifested DNA damage (Bonisoli-Alquati et al. 2018). *γ*-H2AX foci in lymphocytes, as a biomarker for DNA damage, was two times higher in cattle in the Fukushima area captured in 2011 compared to the data obtained in uncontaminated areas. The DNA damage decreased after 2012 and 2013 (Nakamura et al. 2017). The investigation of the hematopoiesis of acute radiation syndrome in Japanese nonhuman primates exposed to radiation in Fukushima is more or less touching the edge of mechanistic elucidation (Urushihara et al. 2018). More studies of this type performed using the state-of-art technology such as epigenomics, genomics, metabolomics, and proteomics should be explored.

In experimental laboratory animal models where radiation doses, radiation dose rates, temperature, air pressure, and water and food availability are controlled, acute radiation exposure results in hematopoietic acute radiation syndrome, gastrointestinal acute radiation syndrome, cutaneous radiation syndrome, cardiovascular syndrome and neurological syndrome depending on the dose of radiation. Changes in DNA damage, microRNA-34a, cytokine/chemokine storms in circulation and tissues, inflammasome formation, inducible nitric oxide synthase (Kiang et al. 2010; Williams et al. 2010), AKT activation, MAPK activation (Wang et al. 2015; Kiang and Olabisi 2019), ATP production, apoptosis (Kiang et al. 2019, 2020), autophagy, and necroptosis (Hei et al. 2011; Kiang and Olabisi 2019) are well characterized and targeted for drug development. Chronic radiation exposure has been less studied, the definition of chronic radiation in the laboratory is temporally limited and the mechanisms studied are also limited (Fuller et al. 2019; Murat El Houdigui et al. 2019; Ojima et al. 2019; Overbey et al. 2019; Koval et al. 2020; Shuryak 2020). A problem developing in one species can have an effect on another species (i.e. interspecies interaction and competition such as deficiency of food chain), possibly struggling species, and that is also difficult to address in the laboratory. Although it is evident that some organisms in their natural environments are more sensitive to radiation than in the laboratory (Garnier-Laplace et al. 2013), investigating the mechanisms underlying that sensitivity is very difficult to do in the wild and nearly impossible to fully model in the laboratory.

### Biomarkers for ecosystems post-irradiation

As far as elucidation of molecular mechanisms, there is also very little investigation into dose biomarkers for wildlife post-irradiation. Biomarkers for experimental animal models in laboratories have been identified for each organ/radiation syndrome and are well established. Table 3 lists biomarkers, in particular those well suited for human triage after a mass casualty event such as a radiation accident or a nuclear detonation. Changes in white blood cells (WBCs), red blood cells (RBCs), platelets, Fms-related tyrosine kinase 3 ligand (Flt-3 ligand), granulocyte-colony stimulating factor (G-CSF), interlukin-18 (IL-18), thrombopoietin (TPO), erythropoietin (EPO) and serum amylase A (SAA) all vary in a radiation dose-dependent manner (Hegge and King 2017; Kiang et al. 2018; Ossetrova et al. 2018a, 2018b). All of them are independent of gender. Except platelets, these are independent of dose rates. Generally, circulating Flt-3 ligand and citrulline/IL-18 concentrations are the biomarkers for hematopoietic syndrome (Kiang and Olabisi 2019) and gastrointestinal syndrome (Bujold et al. 2016; Kiang et al. 2020) respectively. CD31 in skin injury (Doctrow et al. 2013) and ATP production in brain tissues (Kiang et al. 2019) were reported as good biomarkers. It is highly possible that there are similar biomarkers for wildlife species as well. Synergistic effects of combined radiation injury have been documented in survivors from atomic bombing and laboratory animals (Kiang et al. 2010; Kiang and Olabisi 2019). Confounding factors (gender, age, wound, burn, hemorrhage, bone fracture, insecticide, fertilizer, etc.) must be considered when studying biomarkers for the ecosystem after irradiation with time as a key variable. The long-term effects of radiation on changes in the ecosystem will have a profound effect on humans. The knowledge of how to prevent or mitigate radiation or other negative impacts on the ecosystem is likely to be key to human survival.

### Challenges and perspective

There are a vast number of questions about how radiation alters the ecosystem. New techniques and tools available for laboratory experiments are also available for detecting changes in wildlife samples collected from radiation contaminated areas. Major challenges for such work include: (i) gene maps for wildlife are not available, (ii) antibodies against proteins responsible for signal transduction identified in mammals are not available or tested/proved for wildlife, and (iii) systems biology approaches utilized in laboratory experiments are not well developed for studying wildlife. Additionally, if wildlife adaptation is indeed occurring, the collection of samples before adaptation sets in becomes very critical. Collective effort from this research community is imperative to overcome these challenging issues. Ultimately, further funding is required to advance our understanding at the systemic, organismic, cellular, and molecular levels.

## Conclusions

It has been 34 years since the Chernobyl Power Plant accident. The impact of radiation on wildlife is evident, but only few studies have been performed on long-term effects. More observations in a greater variety of species are needed. Nonetheless, it is delightful to detect the presence of adaptation. The wildlife population and abundance are in recovery. However, it would be useful to design experimental models that can be more closely to mimic the natural environment, to aid in elucidating the molecular mechanisms of radiation responses and to establish biomarkers of ecosystems impacted by radiation.

## Figures and Tables

**Table 1.** Factors affecting variability of insect population responses to different doses of irradiation as reported by Moller and Mousseau (2009).

	Location*	Habitat	Soil	Water	Temperature	Cloud Cover	Wind	Time of Day	Date	Year
Bumble-bees	Yes	Yes	No	No	Yes	No	Yes	No	No	Yes
Butterflies	Yes	Yes	Yes	No	No	No	Yes	Yes	No	No
Grasshoppers	Yes	Yes	No	No	Yes	Yes	No	Yes	Yes	No
Dragonflies	Yes	Yes	No	Yes	No	No	No	Yes	No	No
Spider Webs	Yes	No	No	No	Yes	No	Yes	No	No	Yes

* Different sites (there were a total of 700) that had the same radiation levels might have differing habitat (i.e. ground cover), soil composition, or water level/access. Since the collections took place over 3 years, temperature, cloud cover, wind, time of day, actual date and year were also considered and tested as potential confounding variables.

**Table 2.** Brief summary of recent papers that addressed adaptation to ionizing radiation. Papers were varied as to dose levels (and how they were measured), sample sizes, experimental designs and significance levels.

Species	Design	Measured	Adapt.	Area	Reference
Plants					
Evening primrose/*Cenothera biennis*	Field samples	DNA repair	Y	Chern	Boubriak et al. 2008
Thale cess/*Arabidopsis thaliana*	Transplant	Antioxidants	N	Chern	Morozova et al. 2020
	Transplant	MR, Antioxidants, Methylation	Y	Chern	Kovalchuk et al. 2004
Wild carrot/*Daucus carota*	Transplant	Germination	N	Chern	Boratynski et al. 2016
Soybean/*Glycine max*	Transplant	DNA repair, Methylation	Y	Chern	Georgieva et al. 2017
	Field samples	Proteomics comparison	Y	Chern	Danchenko et al. 2009
	Field samples	Proteomics comparison	Y	Chern	Klubicova et al. 2012
Flax/*Linum usitatissimum*	Field samples	Proteomics comparison	N	Chern	Klubicova et al. 2010
White birch/*Betula verrucosa*	Field samples	DNA repair	Y	Chern	Boubriak et al. 2008
Scots pine/*Pinus sylvstris*	Field samples	Antioxidants	Y	Chern	Volkova et al. 2017
	Field samples/2° expo.	Aberrant cells/root meristems	N	Chern	Geras’kin et al. 2011
	Field samples	AFLP-PCR	Y	Chern	Kuchma and Finkeldey 2011
	Field samples	Methylation	Y	Chern	Kovalchuk et al. 2003
Insects					
Fruit flies/*Drosophila melanogaster*	Field samples	SLRL frequencies	Y	Chern	Hancock et al. 2019
Grasshopper/*Chorthippus albomargintus*	Transplant	Development	Y	Chern	Bonisoli-Alquati et al. 2018
	Transplant	Wing size/shape, development	N	Chern	Beasley et al. 2012
Butterfly/*Zizeeria maha*	Transplant/2° expo.	forewing size over generations	Y	Fuku	Hiyama et al. 2015
Birds					
multiple	Field samples	DNA damage, antioxidants	Y		Galvan et al. 2014
Mammals					
Voles; Vole-moles	Field samples	Oxidative stress/Antiox.	Y	EURT	Rasina et al. 2017
	Field samples	Micronuclei/Immunological Indices	Y	EURT	Grigorkina and Olenev 2009
Bank vole/*Myodes glareolus*	FFS/2° expo.	Antiox./ resistance to DNA damage	Y	Chern	Mustonen et al. 2018
Field mice/*Apodemus speciosus*	Field samples	Spermatogenesis	Y	Fuku	Takino et al. 2017

Adapt: adaptation; Antiox: antioxident; Chern: Chernobyl; EURT: East ural radioactive trace; Expo.: exposure; FFS: Fibroblasts from field samples; Fuku: Fukushima; Immunol: immunological; MR: Metabolic Rate; N: No; SLRL: Sex-link recessive lethal; Y: Yes.

**Table 3.** Effects of radiation doses, radiation dose rates and genders on blood cells, cytokines, thrombopoietin, erythropoietin and serum amylase A in blood.

Blood	Rad Dose	Rad Dose Rate	Gender
WBCs	YES	NO	NO
RBCs	YES	NO	NO
Platelets	YES	YES	NO
Flt-3 ligand	YES	NO	NO
G-CSF	YES	NO	NO
IL-18	YES	NO	NO
TPO	YES	NO	NO
EPO	YES	NO	NO
SAA	YES	NO	NO

WBC: white blood cell; RBC: red blood cell; Flt-3 ligand: Fms-related tyrosine kinase 3 ligand; G-CSF: granulocyte-colony stimulating factor; IL-18: interleukin-18; TPO: thrombopoietin; EPO: erythropoietin; SAA: serum amylase A.
